# The 12-Month Prevalence and Trends in DSM–IV Alcohol Abuse and Dependence: United States, 1991–1992 and 2001–2002

**Published:** 2006

**Authors:** Bridget F. Grant, Deborah A. Dawson, Frederick S. Stinson, S. Patricia Chou, Mary C. Dufour, Roger P. Pickering

**Affiliations:** a Laboratory of Epidemiology and Biometry, Division of Intramural Clinical and Biological Research, Department of Health and Human Services, National Institute on Alcohol Abuse and Alcoholism, National Institutes of Health, Bethesda, MD; b Office of the Director, Department of Health and Human Services, National Institute on Alcohol Abuse and Alcoholism, National Institutes of Health, Bethesda, MD

**Keywords:** DSM–IV alcohol abuse and dependence, Epidemiology, Secular trends

## Abstract

**Background:**

Alcohol abuse and dependence can be disabling disorders, but accurate information is lacking on the prevalence of current Diagnostic and Statistical Manual, Fourth Edition (DSM–IV) alcohol abuse and dependence and how this has changed over the past decade. The purpose of this study was to present nationally representative data on the prevalence of 12-month DSM–IV alcohol abuse and dependence in 2001–2002 and, for the first time, to examine trends in alcohol abuse and dependence between 1991–1992 and 2001–2002.

**Methods:**

Prevalences and trends of alcohol abuse and dependence in the United States were derived from face-to-face interviews in the National Institute on Alcohol Abuse and Alcoholism’s (NIAAA) 2001–2002 National Epidemiologic Survey on Alcohol and Related Conditions (NESARC: n = 43,093) and NIAAA’s 1991–1992 National Longitudinal Alcohol Epidemiologic Survey (NLAES: n = 42,862).

**Results:**

Prevalences of DSM–IV alcohol abuse and dependence in 2001–2002 were 4.65 and 3.81 percent. Abuse and dependence were more common among males and among younger respondents. The prevalence of abuse was greater among Whites than among Blacks, Asians, and Hispanics. The prevalence of dependence was higher in Whites, Native Americans, and Hispanics than Asians. Between 1991–1992 and 2001–2002, abuse increased while dependence declined. Increases in alcohol abuse were observed among males, females, and young Black and Hispanic minorities, while the rates of dependence rose among males, young Black females, and Asian males.

**Conclusions:**

This study underscores the need to continue monitoring prevalence and trends and to design culturally sensitive prevention and intervention programs.

## Introduction

Alcohol use disorders are among the most prevalent mental disorders worldwide and rank high as a cause of disability burden in most regions of the world (World Health Organization, 2001). In 2003, the prevalence of alcohol use disorders was estimated at 1.7 percent globally, and these disorders accounted for 1.4 percent of the total world disease burden (World Health Organization, 2003).

Alcohol use disorders (i.e., alcohol abuse and dependence) also are among the most prevalent mental disorders in the United States and are associated with substantial personal and societal costs (Goetzel et al., 2003; Roy-Byrne et al., 2000; Sanderson and Andrews, 2002; Stewart et al., 2003). These alcohol use disorders have enormous consequences not only for the health and welfare of those afflicted with the disorders, but also for their families and children, their employers, and the larger society. For example, approximately one in four children under 18 years old in the United States is exposed to alcohol abuse or alcohol dependence in the family (Grant, 2000). More than one-half of American adults have a family member who has or has had alcohol dependence (Dawson and Grant, 1998). Of the 11.1 million victims of violent crime each year, almost one in four, or 2.7 million, report that the offender had been drinking prior to the crime (Greenfield, 1998). The economic costs of alcohol abuse and dependence were $184.6 billion for 1998 (the last year for which figures are available), or roughly $638 for every man, woman, and child living in the United States (Harwood, 1998). Thus, alcohol use disorders impose a staggering, but potentially preventable, burden.

Despite the importance of accurate prevalence information on alcohol abuse and dependence, especially on changes in the prevalence of these disorders over time, the information available to date has been surprisingly sparse. Time (secular) trends in yearly per capita alcohol consumption have been available from the founding days of the United States. However, these are not informative about whether time trends have occurred in lower or higher consumption levels, which have considerably different public health implications. Time trend data are also available on alcohol-related fatal automobile crashes, but these may reflect factors unrelated to alcohol that are not measured in large statistical reporting systems. Similarly, time trends in alcohol-related liver cirrhosis mortality may reflect the influence of causes other than alcohol. Some papers on time trends in drinking and alcoholrelated problems have been published over the years (Greenfield et al., 2000; Hasin et al., 1990; Midanik and Greenfield, 2000). However, these studies did not cover abuse and dependence as defined in the standard nomenclatures, and were based on very small samples. Thus, trivial changes in numbers from one survey to the next may have given rise to misleading impressions. Finally, three earlier surveys were conducted on alcohol abuse and dependence as defined in the *Diagnostic and Statistical Manual of Mental Disorders* (DSM) of the American Psychiatric Association: the 1980 Epidemiologic Catchment Area Survey (ECA) (Robins and Regier, 1991), the 1990–1992 National Comorbidity Survey (NCS) (Kessler et al., 1996), and the 2001–2002 NCS–Replication Survey (NCS–R) (Kessler and Walters, 2002). However, in addition to major differences in survey designs that preclude clear information about time trends, each of these studies was based on a different nomenclature—*Diagnostic and Statistical Manual, Third Edition* (DSM–III) (American Psychiatric Association, 1980); *Diagnostic and Statistical Manual, Third Edition, Revised* (DSM–III–R) (American Psychiatric Association, 1987); and *Diagnostic and Statistical Manual, Fourth Edition* (DSM–IV) (American Psychiatric Association, 1994)—making between-survey comparisons for the purpose of time trend analysis impossible.

Change or stability in the prevalence of alcohol abuse and dependence has important research and public health implications in a number of areas. For research on the etiology of alcohol dependence (a complex disorder with both genetic and environmental influences), understanding true changes in prevalence over time may be crucial in interpreting familial aggregation and gene–phenotype associations (Rice et al., 2003). Since the distribution of risk or protective genotypes does not vary within a period as short as a decade, changing prevalence suggests changes in the level of environmental risk. Incorporation of this information into the design of genetic studies may sharpen our ability to detect genetic effects. For policy and prevention efforts, accurate information on changes in potentially vulnerable groups may highlight the need for focused planning on both the national and local levels. The fact that accurate data on time trends in the prevalence of alcohol abuse and dependence have not been available reflects a major gap in public health information. The present study was designed, in part, to address this gap and provide the information.

Accordingly, this report presents data on the 12-month prevalence of alcohol abuse and dependence in the United States assessed in the National Institute on Alcohol Abuse and Alcoholism’s (NIAAA) 2001–2002 National Epidemiologic Survey on Alcohol and Related Conditions (NESARC) (Grant et al., 2003b). This report also presents, for the first time, trends in the prevalence of alcohol abuse and dependence between 1991–1992 and 2001–2002 using the NIAAA NESARC and NIAAA’s 1991–1992 National Longitudinal Alcohol Epidemiologic Survey (NLAES) (Grant et al., 1994) as the baseline. The 1991–1992 NLAES and 2001–2002 NESARC are the only two national surveys conducted over the last decade to use consistent diagnostic definitions of alcohol abuse and dependence, both of which were based on the most current psychiatric nomenclature, the DSM–IV.

## Methods

### NESARC Sample

The 2001–2002 NESARC is a representative sample of the United States sponsored by NIAAA that has been described in detail elsewhere (Grant et al., 2003b). The target population of NESARC was the civilian noninstitutionalized population, age 18 and older, residing in the United States and the District of Columbia, including Alaska and Hawaii. The sample included persons living in households, the military living off base, and the following group quarters: boarding houses, rooming houses, nontransient hotels and motels, shelters, facilities for housing workers, college quarters, and group homes. Face-to-face personal interviews were conducted with 43,093 respondents. The fieldwork for NESARC was conducted by the United States Census Bureau. The sampling frame response rate was 99 percent, the household response rate was 89 percent, and the person response rate was 93 percent, yielding an overall survey response rate of 81 percent.

The sampling frame of housing units for NESARC is the Census Supplementary Survey (C2SS), which included 2,000 primary sampling units (PSUs) consisting of all 3,142 counties and county equivalents in the United States. The C2SS sample consisted of 655 PSUs. For NESARC, a sample was drawn from each of the C2SS’s 655 PSUs in addition to a group quarters sampling frame selected from the Census 2000 Group Quarters Inventory. Group quarter units were converted to housing unit equivalents and sampled together with other NESARC housing units. To prevent potential respondent disclosure, smaller PSUs were collapsed so that the final NESARC data file shows 435 PSUs.

Oversampling of Blacks and Hispanics was accomplished at the design phase of the survey. Oversampling increased the proportion of Hispanic and Black households to approximately 20 percent each of the total sample. For each housing unit, one person was selected randomly from a roster of persons living in the household. At this stage in the survey design, young adults (ages 18–24) were oversampled at a rate of 2.25:1.00.

The NESARC data were weighted to reflect the probabilities of selection of PSUs within strata and for the selection of housing units within the sample PSUs. The data were also weighted: (1) to account for the selection of one sample person from each household; (2) to account for oversampling of young adults; (3) to adjust for nonresponse at the household level and person level; and (4) to reduce the variance arising from selecting two PSUs to represent an entire stratum. The weighted data were then adjusted to be representative of the civilian noninstitutionalized population of the United States for a variety of socioeconomic variables including region, age, sex, race, and ethnicity using the 2000 Decennial Census of Population and Housing.

### NLAES Sample

The 1991–1992 NLAES is a nationally representative survey of the United States population sponsored by NIAAA and has been described in more detail elsewhere (Grant et al., 1992, 1994). This survey has been a source of much important information on alcohol use disorders in the United States during the last decade (Grant, 1997a,b, 2000; Grant and Dawson, 1997). Fieldwork for NLAES also was conducted by the United States Census Bureau. Direct face-to-face interviews were administered to 42,862 respondents, age 18 and older, residing in the noninstitutionalized population of the contiguous United States, including the District of Columbia. The household response rate for this survey was 92 percent, and the sample person response rate was 98 percent, for an overall survey response rate of 90 percent.

NLAES featured a complex multistage design. PSUs were stratified according to sociodemographic criteria and were selected with probability proportional to their population size. From a sampling frame of approximately 2,000 PSUs, 198 were selected for inclusion in the NLAES sample. Within PSUs, geographically defined secondary sampling units, referred to as segments, were selected systematically for sample. Oversampling of the Black population was accomplished at this stage of sample selection in order to secure adequate numbers for analytic purposes. Within each household, one randomly selected respondent, age 18 or older, was selected to participate in the survey. Oversampling of young adults, ages 18–29, was accomplished at this stage of the sample selection. This subgroup of young adults was randomly sampled at a ratio of 2.25:1.00.

The NLAES data were weighted: (1) to adjust for probabilities of selection; (2) to account for the selection of one sample person from each household; (3) to account for oversampling young adults; and (4) to account for nonresponse at the household and person levels. Once weighted, the data were adjusted to be representative of the United States civilian noninstitutionalized population for a variety of sociodemographic variables, including region, age, sex, race, and ethnicity using the 1990 Decennial Census of Population and Housing.

### Interviewers and Interviewer Training

Experienced lay interviewers from the U.S. Census Bureau administered the NLAES and NESARC interviews. On average, the 1,000 NLAES and 1,800 NESARC interviewers had 5 years’ experience working on Census and other health-related surveys. NLAES used a paper-and-pencil survey instrument, whereas the NESARC survey instrument was computerized with software that included built-in skip, logic, and consistency checks. The NLAES and NESARC interviewers completed a rigorous 5-day self-study at home and participated in a 5-day in-class training session at one of the Bureau’s 12 regional offices. NESARC training supervisors from each regional office also were required to complete the home study and to attend a centralized training session, where they completed the in-class training under the direction of NIAAA sponsors and Census Field and Demographics Surveys Division Headquarters Staff.

Regional supervisors recontacted a random 10 percent of all the NLAES and NESARC respondents by telephone for quality control purposes and to verify the accuracy of the interviewer’s performance. During this quality control interview, respondents were reasked a set of 30 questions from different sections of the interview to verify their answers. In addition, 2,657 NESARC respondents were randomly selected to participate in a face-to-face reinterview study after completion of their NESARC interview. Each of these respondents was readministered one to three complete sections of the NESARC survey instrument. These interviews not only served as an additional rigorous check on survey data quality, but they also formed the basis of the test–retest reliability study of the new modules of the survey instrument (Grant et al., 2003a). Thus, the field procedures in the two surveys were rigorous and highly similar.

### Diagnostic Assessment

The diagnoses of alcohol abuse and dependence in NLAES and NESARC were made according to the DSM–IV criteria. The diagnostic interview used to generate these diagnoses was the NIAAA Alcohol Use Disorder and Associated Disabilities Interview Schedule–DSM–IV (AUDADIS–IV: Grant et al., 2001), a state-of-the-art structured diagnostic interview designed for use by lay interviewers who are not clinicians. Although the DSM–IV classification was not published until the second quarter of 1994, all of the specific diagnostic criteria for DSM–IV alcohol abuse and dependence (American Psychiatric Association, 1991) were known prior to the fieldwork for NLAES and, therefore, were incorporated into the AUDADIS–IV in their entirety. What was not known at the time was which diagnostic criteria would be assigned to the abuse or dependence categories. However, since all relevant DSM–IV diagnostic criteria had been incorporated into the AUDADIS–IV, computer algorithms were designed to produce diagnoses of abuse and dependence that accurately represented the placement of the criteria within abuse and dependence categories consistent with the final DSM–IV criteria.

DSM–IV alcohol abuse and dependence are defined as maladaptive patterns of drinking, leading to clinically significant impairment or distress. DSM–IV alcohol abuse is manifested by one or more of the following symptoms: recurrent drinking resulting in failure to fulfill major role obligations; recurrent drinking in hazardous situations; recurrent drinking-related legal problems; and continued drinking despite recurrent social or interpersonal problems caused or exacerbated by drinking. DSM–IV alcohol dependence is defined by seven diagnostic criteria: tolerance; the withdrawal syndrome or drinking to relieve or avoid withdrawal symptoms; drinking larger amounts or for a longer period than intended; persistent desire or unsuccessful attempts to cut down on drinking; spending a great deal of time obtaining alcohol, drinking, or recovering from the effects of drinking; giving up important social, occupational, or recreational activities in favor of drinking; and continued drinking despite a physical or psychological problem caused or exacerbated by drinking.

Consistent with the DSM–IV, NLAES and NESARC 12-month diagnoses of alcohol abuse required a respondent to meet at least one of four criteria defined for abuse in the 12-month period preceding the interview and not concurrently meet criteria for DSM–IV alcohol dependence. To be classified with a diagnosis of 12-month alcohol dependence, respondents were required to satisfy at least three of the seven DSM–IV criteria for dependence during the last year. The withdrawal criterion of the alcohol dependence diagnosis was measured as a syndrome, requiring at least two positive symptoms of withdrawal as defined in the DSM–IV alcohol withdrawal diagnosis.

Both the NLAES and NESARC versions of the AUDADIS–IV represented the duration criteria of both the alcohol abuse and dependence diagnoses. The duration criteria relate to specific abuse and dependence criteria, define the repetitiveness with which these diagnostic criteria must occur in order to be positive, and are operationalized by means of qualifiers, such as “recurrent,” “often,” and “persistent.” Duration criteria are not associated with all abuse and dependence criteria. In NLAES, the duration criteria were operationalized by asking respondents if a symptom item had happened more than once. In NESARC, duration criteria associated with abuse and dependence criteria were embedded directly into the symptom questions (e.g., “more than once” want to stop or cut down on your drinking; have “periods” when you ended up drinking more than you meant to).

The NESARC measure of dependence included two new questions, one to measure the “tolerance” criterion, and one to measure the “continued to drink despite persistent or recurrent physical or psychological problems caused or exacerbated by drinking” criterion. The abuse measure included one additional question to operationalize the “hazardous drinking” criterion. To determine the extent to which the addition of these questions might have influenced the comparability of NLAES and NESARC definitions, the questions were removed from the NESARC algorithms used to arrive at abuse and dependence diagnoses, and the associated prevalences were recalculated. Once removed, the NESARC rates of abuse and dependence decreased by 0.30 percent and 0.36 percent. These results indicate that any differences in the operationalizations of alcohol abuse and dependence due to the addition of the new NESARC questions had a trivial impact on the comparability of NESARC and NLAES definitions and were highly unlikely to account for the trends reported here.

The reliability and validity of AUDADIS–IV alcohol abuse and dependence diagnoses are well documented in numerous national and international psychometric studies. These have been conducted in clinical, and particularly in general population samples, the population for which it was designed (Canino et al., 1999; Cottler et al., 1997; Grant, 1992, 1996a, b,c; Grant and Harford, 1989, 1990; Grant et al., 1995, 2003a; Harford and Grant, 1994; Hasin and Paykin, 1999; Hasin et al., 1996, l997a,c,d, 2000; Muthen et al., 1993). Further, the psychometric properties of the AUDADIS–IV alcohol and drug modules were examined in numerous countries in the World Health Organization–National Institutes of Health Joint Project on Reliability and Validity (Chatterji et al., 1997; Hasin et al., 1997b; Nelson et al., 1999; Pull et al., 1997; Ustun et al., 1997; Vrasti et al., 1998).

### Analysis Procedures

As a result of the complex sample design of NLAES and NESARC, specialized software was required to estimate the standard errors of all prevalence estimates. Standard errors presented here were estimated separately for NESARC and NLAES using SUDAAN (Research Triangle Institute, 2002), a software program that uses Taylor series linearization to adjust for complex survey sample design characteristics. For trend analyses, prevalence estimates, and standard errors that were derived separately for NLAES and NESARC were compared using *t*-tests designed for independent samples. In analyses comparing 12-month prevalences among subgroups of the population defined by age, sex, and race–ethnicity using NESARC, significance levels were conservatively adjusted downwards to *P* < 0.01 to account for multiple testing.

## Results

### Prevalence of DSM–IV Alcohol Abuse: 2001–2002

The 12-month prevalence rates, standard errors, and population estimates of DSM–IV alcohol abuse in 2001–2002 are presented in Table 1. Overall, the 12-month prevalence of abuse was 4.65 percent, representing 9.7 million adult Americans.

#### Gender

Overall, rates of DSM–IV alcohol abuse in 2001–2002 were substantially and significantly higher for males (6.93 percent) than females (2.55 percent), a ratio of about 2.72. This large and significant gender difference also was found among Whites, Blacks, and Hispanics. Similar gender differences were found among Asians and Native Americans, although these did not reach statistical significance. The sex differential in the prevalence of DSM–IV alcohol abuse was significant within all age groups of the White, Black, and Hispanic subgroups except for Hispanics age 65 and older, where the gender difference was in the same direction but not significant.

#### Race–Ethnicity

The prevalence of alcohol abuse was significantly greater among Whites (5.10 percent) compared to Blacks (3.29 percent), Asians (2.13 percent), and Hispanics (3.97 percent). When race–ethnicity was addressed by gender, White female rates significantly exceeded those of their Black, Asian, and Hispanic counterparts, but White male rates significantly exceeded only those of Black and Asian males. The prevalence of abuse was significantly greater among Native Americans (5.75 percent) and Hispanics (3.97 percent) compared to Asians (2.13 percent), but when broken down by gender, only the Hispanic male rate (6.21 percent) was significantly greater than the Asian male rate (3.20 percent).

#### Age

Overall, each successively older age group demonstrated decreases in the prevalence of alcohol abuse. All age differences were significant except the decline from ages 18–29 to 30–44, which was significant only for women and Hispanics. When examined within race–ethnic and sex groups, these age differences were less consistently significant. For example, the prevalence of abuse among 30- to 44-year-olds was significantly lower than that of 18- to 29-year-olds only among Hispanic males and Asian females. Rates of abuse for 45- to 64-year-olds compared to 30- to 44-year-olds and rates among the oldest age group (age 65 and older) compared to the 45- to 64-year-old age group were significantly lower among all males, all females, and Whites. Significant declines in prevalence between these age groups were also found when male and female Whites were considered separately. Among Blacks taken as a whole, and among Black males and Black females, the rates of abuse were only significantly lower among the oldest age group compared to the 45- to 64-year-old age group.

### Prevalence of DSM–IV Alcohol Dependence: 2001–2002

The prevalence of 12-month DSM–IV alcohol dependence in 2001–2002 was 3.81 percent, representing 7.9 million Americans (Table 2).

#### Gender and Race–Ethnicity

Although the rates of dependence for the total sample were significantly greater for males (5.42 percent) than females (2.32 percent), a ratio of approximately 2.34, this difference was significant only for Whites, Blacks, and Hispanics when sex differences were assessed within each race–ethnic subgroup of the population. Whites (3.83 percent), Native Americans (6.35 percent), and Hispanics (3.95 percent) had a significantly higher prevalence of dependence than Asians (2.41 percent), but no significant differences were found in the rates of dependence between any of the other race–ethnic groups. There were a number of differences at the subgroup level; for example, at ages 18–29, Whites (10.71 percent) had higher rates of dependence than Blacks (6.03 percent) or Hispanics (6.92 percent). Also, at ages 30–44, Asians (0.44 percent) had lower rates of dependence than all other race–ethnic groups.

#### Age

A significant inverse relationship between rates of dependence across each successively older age group was found for the sample as a whole, and also when males and females were considered separately. The same significant relationship of age to rates of dependence was found for White males and females. In other race-by-gender groups, the prevalence of dependence for Black males was significantly lower among the oldest age group (1.10 percent) compared to the 45- to 64-year-old age group (3.98 percent). Also, the prevalence of dependence for Hispanic males was significantly lower in the 30- to 44-year-old age group (5.33 percent) than in the youngest age group (9.58 percent) and significantly lower in the 45- to 64-year-old age group (2.06 percent) than in the 30- to 44-year-old age group.

### Trends in DSM–IV Alcohol Abuse Between 1991–1992 and 2001–2002

The prevalence of 12-month DSM–IV alcohol abuse significantly increased over the last decade, from 3.03 percent in 1991–1992 to 4.65 percent in 2001–2002 (Table 3). For the total sample, the increases were significant for males (4.67 percent to 6.93 percent) and females (1.51 percent to 2.55 percent) and for each age–gender group except 18- to 29-year-olds.

Between 1991–1992 and 2001–2002, the prevalence of abuse increased among all race–ethnic groups except Native Americans. The increases were significant among Whites, Blacks, and Hispanics. Further, the increases were significant within both genders in each of these three race–ethnic groups.

Among Whites, there was a significant increase in the rate of abuse among all male and female age groups, except 18- to 29-year-olds. Among Black males, there were significant increases in the prevalence of abuse among all age groups. Among Black females, alcohol abuse increased somewhat among all ages, although the increase reached significance only for the 45- to 64-year-old age group. Among Hispanics, the rates of alcohol abuse increased slightly in all age groups from 2001–2002 compared to 1991–1992 for each gender, although the increase reached significance only among males ages 18–29. Among Asians, increases in abuse reached significance only for 18- to 29-year-old females and all persons age 65 and older, although nonsignificant increases occurred in several other age groups. Among Native Americans, a significantly increased rate of abuse was found only among 45- to 64-year-old males.

### Trends in DSM–IV Alcohol Dependence Between 1991–1992 and 2001–2002

The prevalence of DSM–IV alcohol dependence significantly decreased, from 4.38 percent to 3.81 percent, over the past decade (Table 4). Rates significantly decreased among males (6.33 percent to 5.42 percent), but remained relatively unchanged among females. Rates also significantly decreased among Whites (4.35 percent to 3.83 percent) and Hispanics (5.78 percent to 3.95 percent) but remained relatively stable among Blacks, Native Americans, and Asians.

When prevalence rates within subgroups of the population were examined, significant increases in dependence were observed for 18- to 29-year-old Black females (2.14 percent to 3.79 percent) and 18- to 29-year-old Asian males (4.09 percent to 10.22 percent). Significant decreases in the rates of dependence occurred among White males (6.16 percent to 5.41 percent), Whites age 65 and older (0.38 percent to 0.18 percent), 30- to 44-year-old Asian males (3.02 percent to 0.28 percent), Hispanic males (9.40 percent to 5.90 percent), 18- to 29-year-old Hispanic males (15.44 percent to 9.58 percent), 45- to 64-year-old Hispanic males (6.70 percent to 2.06 percent), and all 45- to 64-year-old Hispanics (3.65 percent to 1.23 percent). All other subgroup rates of alcohol dependence remained stable over the past decade, neither increasing nor decreasing.

## Discussion

The total prevalence of 12-month DSM–IV alcohol abuse and dependence in 1991–1992 was 7.41 percent, representing 13.8 million adult Americans. In 2001–2002, this prevalence rose to 8.46 percent, representing 17.6 million adult Americans. Given the harmful effects of alcohol use disorders on the afflicted individuals as well as on those around them and society as a whole, alcohol use disorders continue to represent a substantial public health problem.

The high degree of comparability between NLAES and NESARC definitions of alcohol abuse and dependence made it possible, for the first time, to accurately determine trends in these disorders over the course of a decade. During this time, the prevalence of alcohol dependence significantly declined, from 4.38 percent to 3.81 percent. With regard to specific groups, the prevalence of alcohol dependence decreased over the past decade in males while remaining stable in females, resulting in a some-what smaller current gender difference than had been found previously. Dependence also decreased among Whites and Hispanics. However, in contrast, there was a significant increase in the prevalence of alcohol abuse, from 3.03 percent in 1991–1992 to 4.65 percent in 2001–2002. This increase was apparent in both genders and was especially marked in young Black and Hispanic minorities.

The first-time availability of trend data allows constant as well as emerging high-risk subgroups to be identified with much greater clarity and specificity than in the past. For example, Whites, Native Americans, and males remain high-risk subgroups for abuse in 2001–2002, while rates in the other subgroups increased to approach those of these stably high-risk groups. Among the stable high-risk subgroups for dependence were young adults, especially Whites and Native Americans. A group that showed an especially sharp increase was young adult Asian males, a group that has not previously attracted attention as being at elevated risk for alcohol use disorders. Consistent with earlier patterns, respondents in the youngest age groups (ages 18–29 and 30–44) continue to have the highest prevalence of alcohol abuse and dependence. Taken together, these results highlight the need to strengthen existing prevention efforts and to develop new programs designed with the sex and race–ethnic differentials observed in 2001–2002 in mind. Moreover, these findings underscore the need for early prevention programs among all youth, regardless of sex or race–ethnicity.

Male and female rates of abuse have converged over the last decade. Overall, the male to female ratio for alcohol abuse declined from 3.09 in 1991–1992 to 2.72 in 2001–2002. The narrowing in the gap between male and female rates of abuse also was observed among Whites (2.98–2.55), Native Americans (2.97–1.79), Asians (3.5 1–2.83), and Hispanics (4.77–3.76), but not among Blacks. Although convergence in the rates of abuse was found for most age groups among Whites and Hispanics, it was only observed in the youngest age groups (ages 18–29) among Asians (4.46–1.23) and Native Americans (3.40–2.28).

In contrast, a male–female convergence in the prevalence of dependence was found among the 30- to 44-year-old (2.46–1.91) and 45- to 64-year-old (2.85–2.32) age groups in the total sample, a pattern that also was observed among Whites of those ages. Convergence was found among all Blacks and Hispanics and particularly among 18- to 29-year-old Blacks (3.99–2.31) and Hispanics (5.20–2.49). Over the next decade, the design and implementation of programs targeted at females, especially 30- to 44-year-old Whites and 18- to 29-year-olds of each race–ethnic subgroup, can go a long way in reducing the convergence observed among the male and female rates of alcohol abuse and dependence, that is, in the phenomenon of women’s rates rising to match those of men.

One of the interesting results of the trend analysis was that rates of alcohol dependence had declined slightly, whereas rates of abuse had increased. The decrease in the rate of dependence is not surprising, in that most measures of alcohol consumption have also declined slightly over the same period. The National Health Interview Survey found that the percentage of heavy drinkers fell from 7.9 percent to 7.1 percent, and the percentage of adults who drank 5+ drinks monthly or more often fell from 15.6 percent to 14.4 percent (National Center for Health Statistics, 2002). Earlier data from the National Alcohol Surveys conducted in 1990 and 1995 showed stable rates of drinking, any binge drinking, and frequent binge drinking during that period (Greenfield et al., 2000). Similarly, the National Household Survey on Drug Abuse (Substance Abuse and Mental Health Services Administration, 1995, 2002) found little change between 1992 and 2001 in the rates of any past-month drinking (47.8 percent and 48.3 percent, respectively) and heavy past-month drinking (5+ drinks on the same occasion on each of 5 or more days in the past 30 days, 5.0 percent and 5.7 percent, respectively).

What is surprising, then, is that the prevalence of alcohol abuse increased in the face of slightly declining rates of heavy drinking. One possible factor underlying this could be changes in drinking norms as heavy consumption has declined. With the decrease in heavy drinking, social attitudes may have become more negative toward drinking at levels that were previously accepted. If so, this could have led to increased social/interpersonal conflict about drinking even though overall levels have declined. Since social/interpersonal problems related to drinking constitute one of the DSM–IV criteria for alcohol abuse, such interpersonal conflict would be reflected in the increased prevalence of alcohol abuse. The possibility of such environmental influences on alcohol abuse as distinct from dependence has long been recognized (Rounsaville et al., 1986).

Alternatively, alcohol abuse may be related to complex drinking patterns, not measured in previous trend studies of heavy drinking. Drinking patterns related to the development of alcohol abuse may be quite different from those associated with alcohol dependence. Identifying these drinking patterns is a potentially important area for future research. The observation that alcohol abuse is most prevalent among the youngest age groups underscores the need for continued research on drinking pattern trajectories that, for the most part, are initiated in adolescence.

One of the most striking findings from the trend analysis of 12-month DSM–IV alcohol abuse between 1991–1992 and 2001–2002 was that rate of abuse had not increased among White young adults (ages 18–29) but had increased among minority young adults, specifically Black males, Asian females, and Hispanic males. Similarly, the prevalence of 12-month DSM–IV alcohol dependence significantly increased among Black females and Asian males in the 18- to 29-year-old age group, while no such increases were noted for White, Native American, and Hispanic young adults. It should also be noted the prevalence rates of abuse and dependence among Native American young adults are quite high, even though these rates have not significantly increased over the last decade.

The reasons for the rise of alcohol abuse and dependence among these minority young adults are not known. Recently, researchers have highlighted the impact on drinking patterns, abuse, and dependence of acculturative stress felt by immigrants who are faced with adapting to a new society. Socioeconomic stress experienced by race–ethnic minorities because of inadequate financial resources and limited social class standing, and stress or tensions that minorities experience as a result of discrimination have also been implicated (Al-Issa, 1997). Alternatively, the growing numbers of minority youth attending college may increasingly have exposed them to the risks of heavy drinking commonly noted among college students (U.S. Department of Education, 2001). Other researchers have argued that alcohol advertising targeting Black communities has promoted heavier drinking, particularly of malt liquor, among young adults of this group (Hacker et al., 1987; Herd, 1993). Therefore, achieving an understanding of the changes in prevalence among minority young adults over time will require further research, but is an important public health priority.

What is clear is that no single environmental factor can explain the increases in alcohol abuse and dependence prevalence observed in this study among certain minority subgroups of the population. Numerous environmental factors, including social and economic factors, are all likely to serve as mediators of the observed temporal or secular rate changes. With regard to putative economic factors, there is a clear need for additional studies to ascertain how changes in alcoholic beverage prices, taxes, and availability affect the prevalence of alcohol use disorders among race–ethnic and other subgroups of the population. Historical and cultural factors that shape the life history of various race–ethnic minorities in the United States are potentially equally important in understanding the observed secular changes. Within this context, future research will need to more fully address the extraordinary heterogeneity within race–ethnic groups in the search for the explanations of why rates of alcohol abuse and dependence have increased among some minority young adults as opposed to White young adults. For example, rates of heavy drinking, abuse, and dependence differ vastly among Chinese Americans, Japanese Americans, Cambodian Americans, Korean Americans, and Filipino Americans (Price et al., 2002). Similar differentials have also been found among Mexican Americans, Cuban Americans, and Puerto Rican Americans and among some of the over 300 different tribal and language groups that define Native Americans and Alaska Natives living in the United States (Aguirre-Molina and Caetano, 1994; Beauvais, 1998). Understanding and working effectively with cultural beliefs and practices of individuals from diverse race–ethnic backgrounds will be of paramount importance in the development of successful intervention and prevention programs.

At present, research aimed at identifying independent environmental risk factors for alcohol abuse and dependence is hindered by a limited number of reliable and valid measures of relevant environmental conditions. Once these measures are developed, environmental risk conditions can be studied along with new evidence arising from genetic studies in order to guide the search for environmental factors that might modify specific genetic liabilities and thereby reduce the risk of alcohol use disorders. Understanding the gene–environment interaction also will rely heavily on the sharing of conceptual models and assessment methods across traditional disciplines. In the final analysis, attention to environmental factors that might modify genetic liabilities will lead to more effective prevention and control initiatives in the future.

## Figures and Tables

**Table 1:** Twelve-Month Prevalence of DSM–IV Alcohol Abuse by Age, Sex, and Race–Ethnicity: 2001–2002

Sociodemographic Characteristic	Male			Female			Total		
		
	%	S.E.	Population Estimate^a^	%	S.E.	Population Estimate^a^	%	S.E.	Population Estimate^a^
Total									
Total	6.93	0.28	6906	2.55	0.16	2762	4.65	0.18	9668
18–29	9.35	0.61	2110	4.57	0.39	1041	6.95	0.39	3151
30–44	8.69	0.49	2742	3.31	0.28	1080	5.95	0.31	3822
45–64	5.50	0.43	1719	1.70	0.20	566	3.54	0.25	2286
65+	2.36	0.32	335	0.38	0.11	75	1.21	0.15	410
White									
Total	7.45	0.33	5276	2.92	0.19	2236	5.10	0.21	7511
18–29	10.19	0.81	1405	5.56	0.54	777	7.86	0.50	2182
30–44	10.10	0.63	2166	4.13	0.38	902	7.09	0.40	3068
45–64	5.97	0.51	1425	2.02	0.26	499	3.96	0.30	1925
65+	2.38	0.35	279	0.36	0.10	58	1.21	0.16	336
Black									
Total	5.71	0.58	574	1.41	0.19	182	3.29	0.30	756
18–29	6.92	1.28	166	2.10	0.45	68	4.28	0.67	254
30–44	7.04	0.95	238	1.51	0.30	65	3.95	0.46	302
45–64	4.48	0.74	132	1.25	0.37	47	2.66	0.40	178
65+	1.79	0.59	19	0.12	0.12	2	.78	0.25	21
Native American^b^									
Total	7.47	1.65	157	4.18	1.25	97	5.75	1.02	253
18–29	15.25	5.68	56	6.68	3.34	33	10.35	3.11	89
30–44	7.67	3.00	54	6.52	3.01	49	7.07	2.13	102
45–64	4.85	2.12	39	0.00	0.00	0	2.57	1.14	39
65+	3.59	2.49	8	4.12	4.00	15	3.91	2.63	24
Asian^c^									
Total	3.20	0.79	140	1.13	0.41	53	2.13	0.46	193
18–29	4.77	1.81	63	3.89	1.38	47	4.35	1.25	110
30–44	4.22	1.54	64	0.23	0.22	4	2.18	0.79	68
45–64	1.13	0.78	13	0.20	0.20	4	0.61	0.32	16
65+	0.00	0.00	0	0.00	0.00	0	0.00	0.00	0
Hispanic/Latino									
Total	6.21	0.50	759	1.65	0.23	195	3.97	0.30	953
18–29	9.08	1.07	400	3.04	0.63	116	6.28	0.63	516
30–44	4.88	0.59	219	1.46	0.33	61	3.23	0.37	281
45–64	4.35	0.84	111	0.63	0.36	17	2.43	0.39	128
65+	3.69	1.62	29	0.00	0.00	0	1.56	0.66	29

a Population counts are in thousands.

b Includes American Indians and Alaska Natives.

c Includes Native Hawaiians and other Pacific Islanders.

**Table 2:** Twelve-Month Prevalence of DSM–IV Alcohol Dependence by Age, Sex, and Race–Ethnicity: 2001–2002

Sociodemographic Characteristic	Male			Female			Total		
		
	%	S.E.	Population Estimate^a^	%	S.E.	Population Estimate^a^	%	S.E.	Population Estimate^a^
Total									
Total	5.42	0.21	5397	2.32	0.13	2515	3.81	0.14	7912
18–29	13.00	0.68	2934	5.52	0.40	1256	9.24	0.41	4190
30–44	4.98	0.34	1570	2.61	0.26	851	3.77	0.23	2422
45–64	2.67	0.25	837	1.15	0.17	383	1.89	0.15	1220
65+	0.39	0.11	56	0.13	0.06	25	0.24	0.06	80
White									
Total	5.41	0.26	3832	2.37	0.16	1811	3.83	0.16	5642
18–29	15.10	0.91	2083	6.38	0.57	891	10.71	0.57	2974
30–44	5.13	0.44	1101	2.84	0.33	621	3.98	0.28	1722
45–64	2.56	0.30	611	1.15	0.21	285	1.84	0.18	895
65+	0.32	0.11	37	0.08	0.05	13	0.18	0.05	50
Black									
Total	5.09	0.52	512	2.39	0.31	309	3.57	0.29	821
18–29	8.75	1.53	235	3.79	0.74	124	6.03	0.82	359
30–44	4.40	0.81	149	2.53	0.43	108	3.36	0.42	257
45–64	3.98	0.76	117	1.74	0.54	65	2.72	0.43	182
65+	1.10	0.58	12	0.71	0.47	12	0.87	0.36	23
Native American^b^									
Total	8.38	1.84	176	4.49	1.32	104	6.35	1.17	280
18–29	15.96	5.61	59	8.73	3.62	43	11.83	3.31	102
30–44	10.94	3.43	77	5.77	3.16	43	8.27	2.32	120
45–64	5.11	2.33	41	2.53	1.70	18	3.90	1.48	59
65+	0.00	0.00	0	0.00	0.00	0	0.00	0.00	0
Asian^c^									
Total	3.56	0.74	1.56	1.34	0.49	63	2.41	0.38	218
18–29	10.22	2.33	135	4.27	1.81	51	7.39	1.15	186
30–44	0.28	0.29	4	0.59	0.44	9	0.44	0.27	14
45–64	1.42	0.89	16	0.15	0.14	2	0.72	0.41	18
65+	0.00	0.00	0	0.00	0.00	0	0.00	0.00	0
Hispanic/Latino									
Total	5.90	0.72	721	1.94	0.27	229	3.95	0.44	950
18–29	9.58	1.33	422	3.85	0.60	147	6.92	0.80	569
30–44	5.33	1.04	240	1.65	0.53	69	3.55	0.72	309
45–64	2.06	0.63	53	0.46	0.19	13	1.23	0.31	65
65+	0.85	0.86	7	0.00	0.00	0	0.36	0.36	7

a Population counts are in thousands.

b Includes American Indians and Alaska Natives.

c Includes Native Hawaiians and other Pacific Islanders.

**Table 3:** Trends in the 12-Month Prevalence of DSM–IV Alcohol Abuse by Age, Sex, and Race–Ethnicity: 1991–1992 and 2001–2002

Sociodemographic Characteristic	Male		Female		Total	
		
	NLAES (1991–1992)	NESARC (2001–2002)	NLAES (1991–1992)	NESARC (2001–2002)	NLAES (1991–1992)	NESARC (2001–2002)
Total						
Total	4.67	6.93^a^	1.51	2.55^a^	3.03	4.65^a^
18–29	9.26	9.35	3.83	4.57	6.54	6.95
30–44	4.58	8.69^a^	1.50	3.31^a^	3.02	5.95^a^
45–64	2.38	5.50^a^	0.38	1.70^a^	1.35	3.54^a^
65+	0.55	2.36^a^	0.04	0.38^a^	0.25	1.21^a^
White						
Total	5.09	7.45^a^	1.71	2.92^a^	3.33	5.10^a^
18–29	10.75	10.19	4.83	5.56	7.83	7.86
30–44	5.16	10.10^a^	1.68	4.13^a^	3.41	7.09^a^
45–64	2.55	5.97^a^	0.44	2.02^a^	1.47	3.96^a^
65+	0.52	2.38^a^	0.04	0.36^a^	0.24	1.21^a^
Black						
Total	2.38	5.71^a^	0.73	1.41^a^	1.46	3.29^a^
18–29	3.38	6.92^b^	1.27	2.10	2.44	4.28^a^
30–44	2.54	7.04^a^	1.00	1.51	1.70	3.95^a^
45–64	1.19	4.48^a^	0.02	1.25^a^	0.54	2.66^a^
65+	0.00	1.79^a^	0.00	0.12	0.00	0.78^a^
Native American						
Total	12.80	7.47	4.31	4.18	8.14	5.75
18–29	27.88	15.25	8.19	6.68	17.59	10.35
30–44	2.64	7.67	3.73	6.52	3.22	7.07
45–64	0.00	4.85^b^	0.00	0.00	0.00	2.57^b^
65+	0.00	3.59	0.00	4.12	0.00	3.91
Asian						
Total	1.65	3.20	0.47	1.13	1.08	2.13
18–29	3.30	4.77	0.74	3.89^b^	2.02	4.35
30–44	1.67	4.22	0.21	0.23	0.93	2.18
45–64	0.00	1.13	0.66	0.20	0.30	0.61
65+	0.00	0.00	0.00	0.00	0.00	0.00
Hispanic/Latino						
Total	4.15	6.21^b^	0.87	1.65^b^	2.52	3.97^a^
18–29	5.85	9.08^b^	1.56	3.04	3.71	6.28^a^
30–44	3.30	4.88	0.86	1.46	2.15	3.23
45–64	3.08	4.35	0.13	0.63	1.60	2.43
65+	2.90	3.69	0.00	0.00	1.16	1.56

a *P* < 0.01.

b *P* < 0.05.

**Table 4:** Trends in the 12-Month Prevalence of DSM–IV Alcohol Dependence by Age, Sex, and Race–Ethnicity: 1991–1992 and 2001–2002

Sociodemographic Characteristic	Male		Female		Total	
		
	NLAES (1991–1992)	NESARC (2001–2002)	NLAES (1991–1992)	NESARC (2001–2002)	NLAES (1991–1992)	NESARC (2001–2002)
Total						
Total	6.33	5.42^a^	2.58	2.32	4.38	3.81^a^
18–29	12.81	13.00	6.01	5.52	9.40	9.24
30–44	6.07	4.98^b^	2.47	2.61	4.25	3.77
45–64	3.19	2.67	1.12	1.15	2.12	1.89
65+	0.63	0.39	0.23	0.13	0.39	0.24
White						
Total	6.16	5.41^b^	2.67	2.37	4.35	3.83^b^
18–29	13.59	15.10	7.28	6.38	10.48	10.71
30–44	6.12	5.13	2.43	2.84	4.27	3.98
45–64	2.80	2.56	1.03	1.15	1.89	1.84
65+	0.57	0.32	0.24	0.08	0.38	0.18^b^
Black						
Total	5.86	5.09	2.21	2.39	3.84	3.57
18–29	8.54	8.75	2.14	3.79^b^	5.07	6.03
30–44	6.13	4.40	3.26	2.53	4.57	3.36
45–64	4.05	3.98	1.95	1.74	2.89	2.72
65+	0.83	1.10	0.00	0.71	0.32	0.87
Native American						
Total	11.00	8.38	7.38	4.49	9.01	6.35
18–29	13.34	15.96	14.67	8.73	14.03	11.83
30–44	8.85	10.94	2.15	5.77	5.32	8.27
45–64	4.61	5.11	4.73	2.53	4.68	3.90
65+	25.39	0.00	0.00	0.00	9.75	0.00
Asian						
Total	3.06	3.56	1.41	1.34	2.26	2.41
18–29	4.09	10.22^b^	3.77	4.27	3.93	7.39
30–44	3.02	0.28^b^	0.00	0.59	1.50	0.44
45–64	2.90	1.42	0.73	0.15	1.90	0.72
65+	0.00	0.00	0.00	0.00	0.00	0.00
Hispanic/Latino						
Total	9.40	5.90^a^	2.15	1.94	5.78	3.95^b^
18–29	15.44	9.58^b^	2.97	3.85	9.21	6.92
30–44	6.56	5.33	2.70	1.65	4.74	3.55
45–64	6.70	2.06^b^	0.63	0.46	3.65	1.23^b^
65+	0.00	0.85	0.62	0.00	0.37	0.36

a *P* < 0.01.

b *P* < 0.05.
